# Optimization of Spatial Pattern of Land Use: Progress, Frontiers, and Prospects

**DOI:** 10.3390/ijerph19105805

**Published:** 2022-05-10

**Authors:** Changchang Liu, Chuxiong Deng, Zhongwu Li, Yaojun Liu, Shuyuan Wang

**Affiliations:** College of Geographic Sciences, Hunan Normal University, Changsha 410081, China; liucc@hunnu.edu.cn (C.L.); lzw17002@hunnu.edu.cn (Z.L.); 202001026@hunnu.edu.cn (Y.L.); Wsy526@smail.hunnu.edu.cn (S.W.)

**Keywords:** land systems, ecological restoration, ecosystem services, conceptual model, transformation threshold

## Abstract

Due to high-intensity human disturbance and rapid climate change, optimizing the spatial pattern of land use has become a pivotal path to restoring ecosystem functions and realizing the sustainable development of human–land relationships. This review uses the literature analysis method combined with CiteSpace to determine current research progress and frontiers, challenges, and directions for further improvement in this field. The main conclusions include the following: (a) research on the optimization of spatial pattern of land use has transformed from pattern description orientation to sustainable development orientation to ecological restoration orientation. Its research paradigm has changed from pattern to function to well-being; (b) the research frontier mainly includes spatial pattern of land use that takes into account the unity of spatial structure and functional attributes, the ecological mechanism and feedback effect of change in spatial pattern of land, the theoretical framework and model construction of land use simulation and prediction based on multiple disciplines and fields, and the adaptive management of sustainable land use in the context of climate change; (c) based on current research challenges, we integrate the research on landscape ecology and ecosystem service flows to develop an “element sets–network structure–system functions–human well-being” conceptual model. We also propose the strengthening of future research on theoretical innovation, spatiotemporal mechanism selection, causal emergence mechanism, the transformation threshold, and uncertainty. We provide innovative ideas for achieving sustainable management of land systems and territorial spatial planning with the aim of improving the adaptability of land use spatial optimization. This is expected to strengthen the ability of land systems to cope with ecological security and climate risks.

## 1. Introduction

Rapid global population growth, urbanization, and industrialization have resulted in the arrival of the “Anthropocene,” a critical period in which human activities are dominating and changing the boundaries of the earth system, driving environmental changes at the surface [1,2]. The current scope, intensity, and impact of global land use are unprecedented in the earth’s history [3]. The spatial land use change has had a profound impact on the structure and function of terrestrial ecosystems and has caused a series of ecological and environmental problems at the regional and global levels. In response to human-induced ecosystem degradation, biodiversity loss, and climate change, the United Nations has declared 2021–2030 the decade of ecosystem restoration [4]. This makes the spatial optimization of land use not only an important segment of global climate and environmental change research but also the core of various sustainable development studies [5,6,7].

Land is an essential spatial carrier of human social and economic activities and ecological civilization construction. The characteristics changes in spatial pattern of land use directly reflect the influence of human activities on the earth’s land surface natural ecosystem. There exists a causal chain of “elements, structure, and function” that leads to ecosystem structure and function changes and indirectly affects the material cycle and ecological processes, such as biodiversity and climate change [8,9]. The non-linear and complex interactions between land use, ecosystems, and socio-economics drive human understanding of human–land relationships from a land systems perspective and guide rational land use to achieve sustainable development [10,11]. The spatial optimization of land use is to adjust the rationality of the quantitative structure of the distribution of regional land resources and human social and economic activities and the spatial layout in accordance with the principles of population, resource, and environmental balance and economic, social, and ecological benefits. It is also to coordinate human activities and protect the ecological pattern and natural resources to achieve the sustainable development of the human–land system [12,13]. With the continuous expansion of the intensity of human interference and the intensification of the impact of global climate change, the actual development and challenges put forward higher requirements for optimizing spatial land use. How to rationally allocate spatial land use and maintain and improve the functions of the land ecosystem against the background of increasingly tense human–land relationships and ecological degradation has become an important issue. Judging from the current research, significant progress has been made in land change monitoring, land system changes and its driving factors analysis, land use transformation, ecological effect evaluation, and land use simulation prediction [14,15,16,17]. However, the connotation and research framework of spatial land use are not unified, and its definition paradigm lags behind the actual development scenario. The driving mechanism of the human–land interaction coupling of changes in spatial land use needs to be strengthened. In addition, the uncertainty in the spatial simulation and prediction of land use at different scales and regions is still a complex problem, and there are still some challenges in system integration and comprehensive analysis [14,18].

With the intensification of ecological degradation, the land system, which is a fundamental component of the earth’s surface, has entered a potential high-risk zone [14]. Spatial optimization of land use has become a critical way to improve the function of land ecosystems and thus promote the sustainable development of coupled human–land systems. Summarizing the existing research hotspots and problems based on an in-depth understanding of the progress of research in this field is essential to explore how to rationalize spatial land use in order to sustainably develop land systems.

For the above reasons, the objectives of this paper are to summarize the development and characteristics of land space optimization research, to determine the frontier issues in this field, and to propose the current challenges and solutions. The purpose of this review is to provide a reference for further development of land system science and sustainable land system management.

## 2. Data and Methods

**Figure 1:**
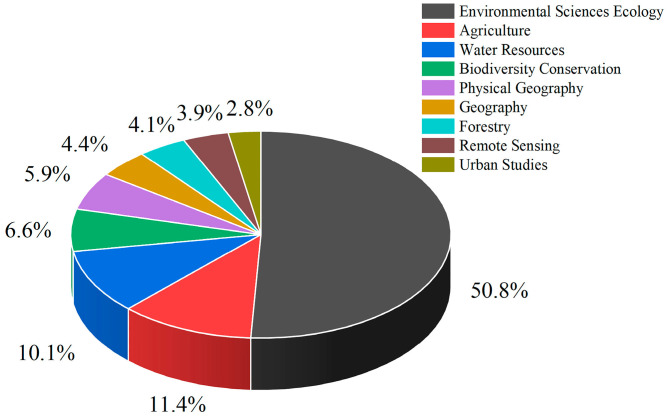
Classification of the articles reviewed in this study by field and their relative proportions.

**Figure 2:**
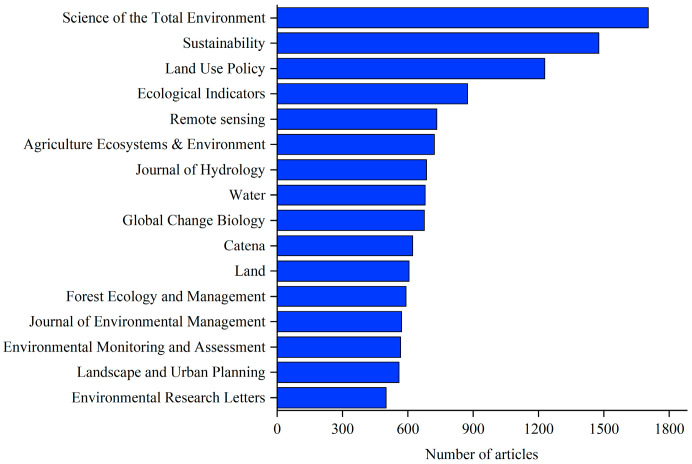
Top ten journals for article publication reviewed in this study.

The data for this study were obtained from the Web of Science core collection database (https://www.webofscience.com/wos/woscc/basic-search, accessed on 27 April 2022). Web of Science is an important database for obtaining global academic information. It includes more than 12,000 authoritative and high-impact academic journals from around the world, covering fields in the natural sciences, engineering technology, biomedical science, and the social sciences [16]. In the Web of Science core collection database search, the language was set to English, the type of document was set to thesis or review paper, and the search topic was set to TS = (“land use”) AND TS = (“change” OR “changes” OR “space”). A total of 61,067 articles were retrieved. As mentioned above, this review focuses on the rational allocation of spatial land use and the maintenance and improvement of land ecosystem functions in the context of increasing human–land tension and ecological degradation. This requires a scientific analysis of the structure, function, and operating mechanisms of the land system. Thus, we excluded articles from unrelated research areas, such as evolutionary biology, material science, physiology, and retained articles from the environmental sciences, ecology, and geography (Figure 1). After excluding unrelated articles, a total of 48,730 articles were obtained, which represent more than 80% of the 61,067 retrieved documents. In terms of journal types (Figure 2), the top ten journals were mainly dominated by ecology, land system science, geography, and environmental science. Since only data from 2005 to 2021 were retrieved from the Web of Science core collection database, articles from 1990 to 2004 were supplemented in the Web of Science database according to the above settings. For avoiding duplicate documents, the duplicate data removal function of CiteSpace 5.8.R3 was used to check the documents, and finally, a total of 49,371 articles were obtained.

## 3. Results

### 3.1. Trend Analysis of Literature Publication

**Figure 3:**
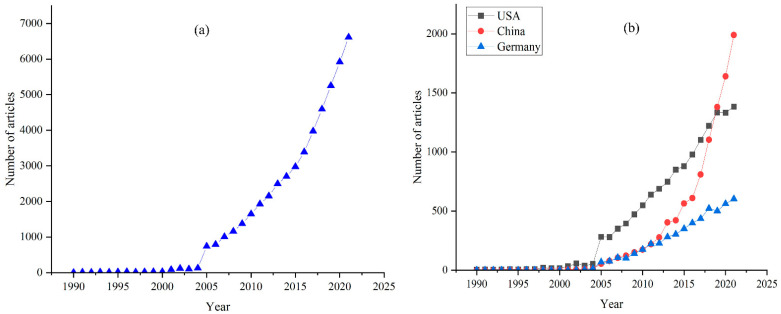
Statistics of selected articles. (**a**) Number of land use change articles published by year from 1990 to 2021, (**b**) Number of articles published by the three countries with the highest number of academic articles published on land use change, 1990–2021.

We found that there were very few articles published on this topic from 1990 to 2005, but a rapid increase in publication took place from 2005 onward (Figure 3a). In terms of place of publication, the top ten countries are the USA, China, Germany, England, Australia, Canada, Spain, the Netherlands, France, and Brazil. By comparing the trends of the three top-ranked countries (Figure 3b), it can be seen that research in Europe and North America started earlier, but its acceleration in terms of publication volume has been less remarkable than that of China. Although China published little in the 1990s, the acceleration in its publication volume has been more remarkable than that of the USA or Germany, and it has surpassed the USA in the number of publications in recent years.

### 3.2. Network Analysis of Cooperation between Countries, Institutions, and Authors

**Figure 4:**
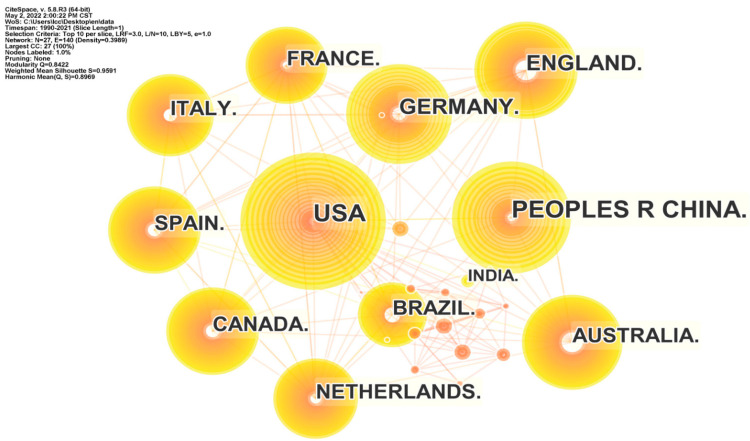
Map of the high–yield country cooperation network.

**Figure 5:**
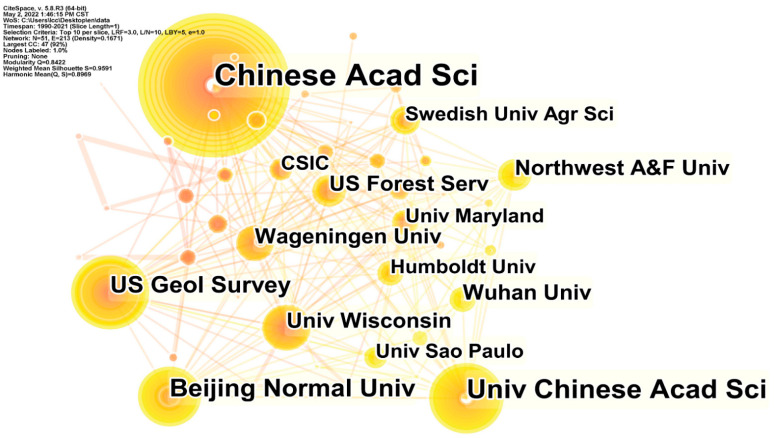
Map of the high–yield institution cooperation network.

**Figure 6:**
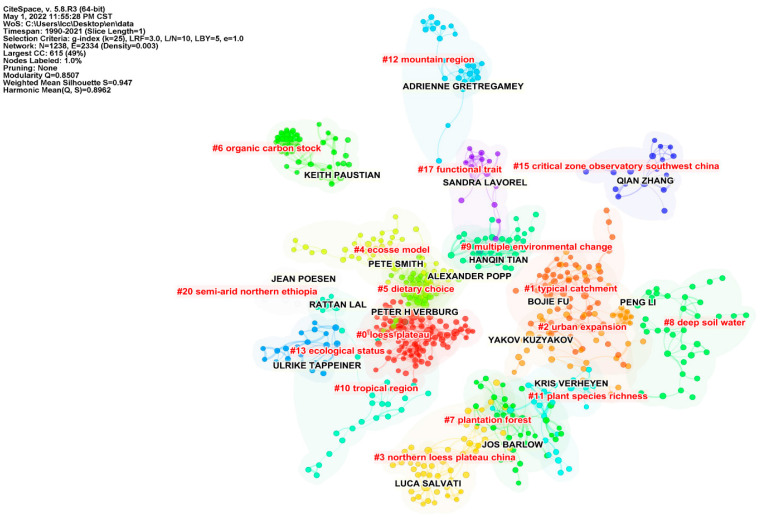
Map of the high–yield author cooperation network.

We analyzed the data structure by countries, institutions, and authors; the threshold in CiteSpace was set at top 10, which captures the top 10 countries, institutions, and authors with the highest frequency of all publications per year. The number and size of nodes in the network mapping reflect the frequency of co-occurrence, and the number and thickness of connection represent the relationship and strength of cooperation, respectively. From the country cooperation network mapping (Figure 4), a network structure with the previously mentioned top 10 articles published countries as the core nodes is obtained. The network of cooperation between countries has 27 nodes and 65 links, with a network density of 0.1852, indicating that the overall cooperation relations between countries in this field are not strong. The tightness of cooperation between countries shows a high regional character. The publishing institutions are characterized by closer cooperation within countries (Figure 5). For example, a high intensity of cooperation is observed between the Chinese Academy of Sciences, the University of Chinese Academy of Sciences, and Beijing Normal University. The network analysis considering “authors” shows predominantly inter-country cooperation (Figure 6). Authors with the highest frequency of publications have developed distinctive research characteristics and formed their own research cores. The overlap in the study topics by different authors also indicates the significant interdisciplinary nature of the field.

### 3.3. Analysis of Keywords and Stages of Development

**Figure 7:**
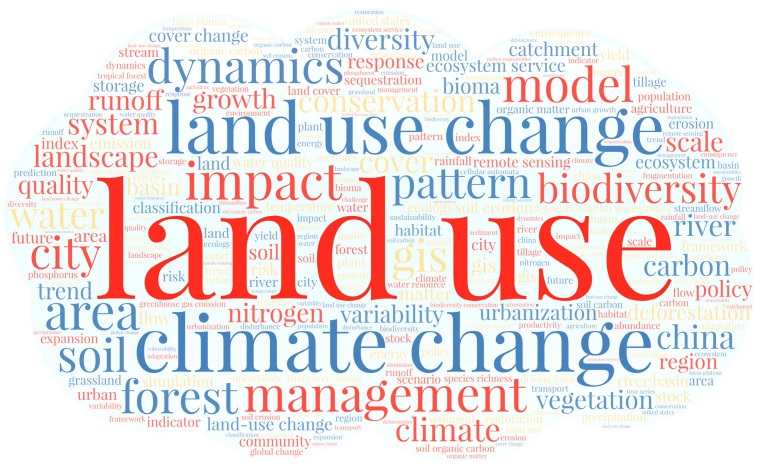
Word cloud of the most frequently used keywords in selected articles.

To better characterize the trends in land use change research, we used CiteSpace 5.8.R3 software to perform word frequency analysis on the keywords of selected articles. The WordArt platform was used to create word cloud maps for keywords that appeared more than 500 times (Figure 7). The top 10 most frequently used words were “land use”, “climate change”, “land use change”, “impact”, “management”, “dynamics”, “biodiversity”, “model”, “conservation”, and “pattern”.

**Figure 8:**
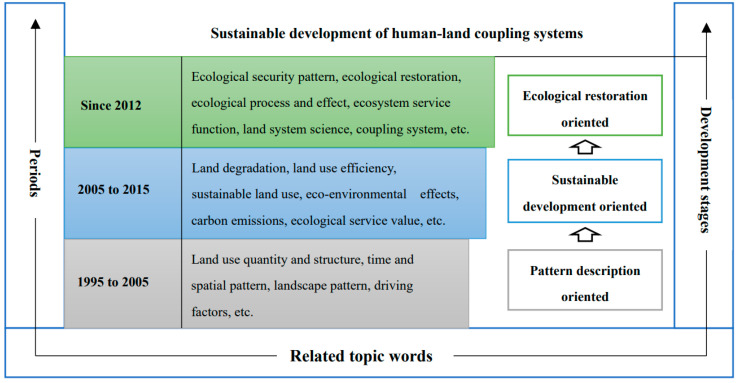
Stages and themes of spatial pattern of land use research.

Through the above literature statistics and keyword analysis, we found that the research on spatial optimization of land use is closely related to the launch of international scientific programs in this field. For example, the International Geosphere-Biosphere Programme (IGBP) and the International Human Dimensions Programme on Global Environmental Change (IHDP) jointly launched the Land-Use and Land-Cover Change (LUCC) project in 1995. Since the implementation of the project, land use change has been a hot issue in international geography research [19]. The IGBP and IHDP also launched the Global Land Programme (GLP) in 2005. The GLP program focuses on coupled human–environment systems, emphasizing integrated and simulation studies of coupled human–environment systems in terrestrial systems [20]. In 2012, the United Nations Conference on Sustainable Development put forward the Future Earth plan, which concentrates on the interrelationship and interaction among human and natural factors, human well-being, and global environmental change [21]. Research on land use change has gradually formed the paradigm of land system science [13,15,22]. Based on the implementation of the above three programs and the analysis of the existing related subject terms, we divided the research on spatial optimization of land use into three stages (Figure 8). It can be seen that with the continuous advancement of research technologies and methods, the research on spatial optimization of land use has continued to deepen, presenting the following transformational trends: the understanding of the connotation of land system had a paradigm shift from focusing on a single land element to the human–land relationship system integrating multiple social and ecological elements; the analysis of the driving force of the process of land use change has deepened to the social and ecological response and mutual feedback mechanism; the evaluation and management of the effects of land use have shifted from focusing on short-term economic benefits to taking into account and adapting to the transformation of the ecological function system in the long-term development and protection.

### 3.4. Research Progress

#### 3.4.1. Analysis of Spatial Measures and Drivers of Land Use

**Figure 9:**
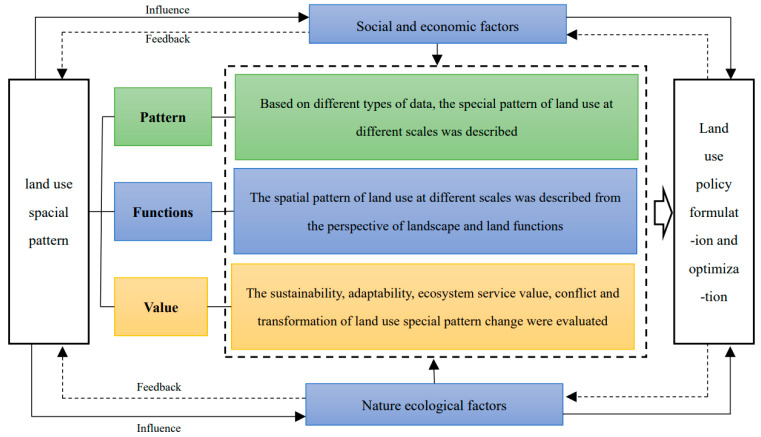
Transformation of spatial pattern of land use optimization.

Measuring and understanding the laws of spatio-temporal changes in land use and influencing factors is the basis of optimizing the spatial pattern of land use. The spatial pattern of land use results from the different development intensities of human social and economic activities under the joint action of internal physical geographical background and external environmental changes in a specific time and region. It has a high degree of spatio-temporal dynamics. Meanwhile, it also has a dimensional nature: space, time, and development intensity [23]. Mapping of spatial patterns is mainly conducted using tools such as geographic information systems (GIS) based on different types of land use data. The commonly used ones are remote-sensing image data and land use survey data. LUCC-based data has benefited from the continuous development of remote-sensing technology, classification systems, and classification methods. It features various resolutions covering the whole world that have been established and improved [18]. In the description of spatial patterns, land use intensity is mainly measured based on different types of data, of which remote-sensing image data and land use survey data are commonly used. The hot spots of concern are concentrated in areas with prominent human–land conflicts and fragile ecological environments, such as urban centers, oases, agro-pastoral ecotones, estuarine deltas, and typical watersheds. [24,25]. The change in landscape pattern is a direct reflection of the change in the spatial pattern of land use; thus, identifying and optimizing the change in spatial pattern of land use from the perspective of landscape pattern is important [26,27]. The connotation scale of spatial description also reflects the ecological attribute characteristics of land use and the requirements of good management. The research on the spatial optimization of land use based on “production–living–ecological” space is a typical representative and has become the theoretical basis for land use development and protection practices [28]. In addition, spatial pattern of land use is the primary carrier and representation of human–land system interaction; the research in this aspect also contains the evaluation of the characteristics of land use development from different perspectives, including the evaluation of sustainability, suitability, efficiency, versatility, conflict, and transformation of land use [29,30,31,32,33]. In general, the study on the measurement of changes in the spatial pattern of land use has undergone a transformation from single-source data to multi-source data, and from a research perspective, it has transformed from pattern to function to value dimension (Figure 9).

The spatial pattern of land use is a complex human–land coupling system formed by the interaction between the ecosystem and human society [23,34]. From this perspective, spatial changes in land use are determined by a combination of natural and socio-economic factors and their interactions. In terms of the driving factors, mainly experience-based statistical models, system dynamics models, or comprehensive models are carried out from the aspects of natural conditions, climate change, economic development, social environment, and population changes [17]. Some scholars have analyzed the influencing factors of land use spatial pattern change from the micro factors, such as commuting time, housing price, and living facilities [35]. Although human social and economic activities are considered the leading factors in the change of spatial pattern of land use, in terms of the background of land as a natural resource and ecosystem, the change of land ecosystem caused by human factors will also have an impact on the spatial pattern of land use. Therefore, in the analytical paradigm of driving factors, we gradually turn to pay equal attention to the “expansion effect” caused by rapid economic growth and the “restraint effect” caused by eco-environmental constraints [36]. It should be noted that land is one of the three factors of production along with labor and capital. The relationship between different scales, institutional structures, socio-economic contexts, subjects of interest, and decision-making processes of land system models is a deeper cause of spatial changes in land use [10]. In reality, these factors have a more profound impact on spatial changes in land use. The national land use regulatory models and legal systems largely influence land expansion, such as specific national and subnational regulations on land use zoning or logging rates (e.g., sugarcane zoning and forestry regulations land reserve quotas in Brazil) [37]. Land management regulations and land tenure rights are closely related to the number and location of transitions in short- and medium-term spatial land use change [38]. However, where institutional capacity is lacking, the contradiction between national development and poorly functioning governance and justice systems can undermine the effectiveness of land regulatory systems. In the case of controlling illegal logging in the context of mitigating CO_2_ emissions, for example, nominal protection has been ineffective in changing the behavior of companies and communities involved in deforestation in Indonesia, and similarly ineffective in controlling deforestation caused by small farmers fleeing conflict areas in Congo [37,38,39]. In addition, there is not only a growing interest in policy change factors, such as agricultural policy reform, trade liberalization, and nature conservation, but also fundamental changes in energy policy and new measures related to climate change adaptation and mitigation [40].

#### 3.4.2. Ecological Effects of Spatial Changes in Land Use

As the key interface linking the four major layers of the earth’s atmosphere, hydrosphere, biosphere, and lithosphere, the change in spatial pattern of land ecosystem involves and affects a series of ecological elements and processes, such as atmosphere, soil, water, and organisms [41]. The first concern is changes in ecological processes and the effects of single and combined elements brought about by spatial changes in land use, which typically involve changes in the atmosphere, water, soil, and biodiversity [42,43,44,45]. Another main topic on the ecological effects of changes in the spatial pattern of land use is ecosystem services. Nature provides food, clean water, healthy soil, carbon capture, and other services for human society. Human well-being depends entirely on the continuous provision of these ecosystem services [46]. Ecosystem services are related to ecosystems and human well-being and can be an essential tool for making spatial optimization land use more in line with the concept of ecological civilization. Using ecosystem services as the carrier to optimize the spatial pattern of land use is conducive to realizing the value of all elements of natural resources and has important practical significance in improving the overall quality of ecosystem functions [47]. The primary goal is to optimize the spatial pattern of land use to achieve the best possible balance between supply and demand for ecosystem services. Related studies mainly take the assessment of ecosystem services as an essential basis for ecological spatial identification, ecological network construction, or optimization of spatial pattern of land use [48,49] according to the ecosystem service tradeoff or collaborative relationship and supply and demand patterns [50,51].

Spatial optimization of land use is important for ecological security; related studies involve the assessment of ecological risk, ecological sensitivity, ecological vulnerability, and ecological safety. As the foremost measure of ecological restoration and protection of the land surface system, the ecological construction project can rapidly change the regional land use pattern to a great extent. The evaluation of spatial patterns of land use and its function in implementing ecological engineering is also considered imperative by scholars [52]. In general, in terms of the evaluation and research of the ecological effects of the spatial pattern of land use, a notable feature is the addition of ecological constraints, economic constraints, social constraints, land applicability constraints, and technical constraints at the front or end of the spatial pattern change to evaluate single or comprehensive ecological spatial effects of land use pattern changes [53]. The research scale includes both macro and micro aspects, especially the studies combining it with ecosystem services, greatly expanding the research connotation and space of the ecological effect evaluation of spatial pattern of land use.

#### 3.4.3. Simulation and Prediction of Spatial Changes in Land Use

Simulating and predicting the dynamics of land systems is a powerful tool for analyzing the structure and function of land use systems, as well as a critical link in understanding the environmental effects of human activities. It provides a scientific basis for formulating land use policies [54]. Land use optimization includes the composition of each land use type (the number of land use types and the proportion of each type) and allocation (the spatial distribution of land use types), which requires a complex tradeoff between multiple land use objectives (such as ecological protection and economic growth) [55]. Therefore, models for integrating these multiple spatial objectives and algorithms for solving spatial optimization problems become vital links. With the development of computer and artificial intelligence technology, spatial simulations and prediction models of land use have been greatly developed. In terms of the types of models, different scholars have different classifications. Zhang et al. divided the spatial simulation and prediction models of land use into spatial models, planning models, simulation and prediction models, intelligent models, hybrid or coupled models, and newly developed models [56]. Different models have different advantages and limitations [57] (see Table 1). Simulation and prediction must seek the best solution in balancing multiple sustainable development goals, such as economic development, ecological protection, and social justice. In this process, ecological elements and ecological processes are receiving more and more attention [58,59,60]. In addition, the combination of different simulations and the development of new models have compensated for the shortcomings between different models and have tried to bridge the gap between research results and practical applications by enhancing their scientific nature and ease of operation [61,62,63,64,65].

In the simulation prediction, different scenarios are set according to the region’s natural endowment and development characteristics, providing diverse options for government management to weigh different spatial patterns of land use. Current scenarios can be broadly classified into four categories: development scenarios, parameter scenarios, climate change scenarios, and ecological restoration scenarios. Development scenarios include rapid economic development, ecological protection, and comprehensive development [66]; parameter scenarios are mainly based on model parameter settings; climate change scenarios mainly consider the impact of climate change; and ecological restoration scenarios are mainly based on the impact of ecological restoration measures on spatial changes in land use, such as setting up chemical fertilizer reduction, riparian buffer zones, returning farmland to forests, and comprehensive restoration [67]. In general, land use simulation research has also shifted from quantitative to spatial pattern simulation and from single-model to coupled-model simulation, showing the development direction of multiple models, multiple technologies, and artificial intelligence. In simulation and prediction, the weight of ecological factors is increasing slightly, and the tradeoff path under the win–win goal of ecological protection and economic development in scenario setting is much more specific. The early warning and the actual reference value of the results have become significant concerns.

### 3.5. Research Frontiers

#### 3.5.1. Measuring Spatial Changes in Land Use with Both Quantity and Quality

Quantitative characterization of global multi-scale change in spatial pattern of land use is always the fundamental basis and core research direction of optimization of spatial pattern of land use. Spatial changes in land use require the discovery and analysis of underlying, meaningful regularities in the seemingly disordered distribution of land use elements and a determination of the mechanisms that generate and control space [68]. Therefore, objectively selecting appropriate indicators and comprehensively describing the spatial pattern from the two aspects of “quality” and “quantity” is an imperative research topic on spatial patterns of land use. Theories of land use versatility, ecosystem services, and landscape function have become important theories for the comprehensive description of land use spatial patterns [69]. This research mainly involves using more accurate data or comprehensive data sets to carry out the quantitative description of the spatial pattern of land use [70] and investigating new mining methods for identifying spatial pattern of land use. For example, Tu et al. proposed a method for mining the spatial pattern of land use using the adaptive adjacency criterion, which combines geographic zoning and adaptive criteria to determine different adjacency thresholds for each zone; the method can determine the spatial dependence between different land use categories [71]. At present, spatial measurement of land use mostly employs remote-sensing data. Although this can reveal land types and usages to a certain extent, the description of socio-economic attributes of land systems still faces technical bottlenecks. On the one hand, in terms of data reliability and accuracy, due to the incompleteness of historical data and the spatial resolution of remote-sensing images, there is always a certain degree of uncertainty and error in the quantitative measurement of spatial changes in land use. On the other hand, the use of remote sensing based on characterization and analysis of biophysical parameters is not sufficient to comprehensively understand changes in the socio-economic functions of land. In particular, the inclusion of socio-economic inputs, ecological impacts, and changes in land management dynamics remains a challenge [10,72,73]. Therefore, in the actual research, it is a significant trend to combine the resolution with the characteristics of the research object, the spatio-temporal background, and the field observation data [74].

#### 3.5.2. Ecological Mechanism and Mutual Feedback Effects

Spatial changes in land use not only directly affect the surface cover but also profoundly affect ecological processes, such as material cycling and energy exchange in the ecosystem. This changes the structure and function of ecosystem elements and leads to a feedback effect on the changes [74,75]. Nowadays, the ecological components concerned with evaluating the ecological effects of changes in the spatial pattern of land use are more systematic, and the comprehensive ecological effect evaluation related to the atmosphere, water, soil, and ecological diversity is the focus of research. Among them, research hotspots mainly include the combined effects of biogeographical and chemical effects of changes in land use under climate change and the process of soil quality and its ecological functions and ecological effects [76,77]. Related hot topics also include landscape ecological patterns and the supply and demand for ecosystem services [78]. In particular, ecosystem services can not only reflect changes in ecological elements caused by land use but also provide a basis for evaluating the ecological effects of spatial pattern changes, accurately identify and quantify the supply, demand, and flow of ecosystem services, and build a research framework of “factor carrier-flow path movement-function enhancement”, which can provide a more scientific basis for the systematic optimization of land spatial patterns [79]. The research in this area focuses more on the use of coupling and dynamic simulation models. It strengthens the human–natural driving mechanism to better understand the “black box” between the spatial pattern of land use and ecological effect.

At the same time, the way in which changes in the spatial pattern of land use affect ecosystem services and the mechanisms by which they affect social and economic development has also become a major issue. It can be seen that the study of land use has shifted from a pure dynamic framework to a paradigm of natural ecological evolution. Integrating ecological knowledge to investigate the laws of ecological evolution of changes in land use emerged as a key trend in optimizing spatial patterns of land use under the guidance of ecological restoration. In this context, the network is the essence of the interaction of various components in the ecosystem and the flow of material and energy. Achieving stability and sustainability based on the structural characteristics of the regional ecological network has become an urgent issue to be solved in the optimization of the spatial pattern of land use [80,81].

#### 3.5.3. The Theoretical Framework and Model Construction of Spatial Simulation and Prediction Based on Interdisciplinary and Technological Integration

The main problems of land use spatial pattern optimization include the following: (1) What is the total income and opportunity cost of each optimization scheme of land use? (2) Which type of land use needs to be allocated for each site? (3) What is the area to be allocated for each land use type, and what are the relevant upper and lower limits for these land uses? (4) What is the effect of increasing or decreasing the area of each land use? (5) In addition to the best type of land use, what is the opportunity cost of land use regulation allocated to a particular location [65]? To solve these problems, we need a systematic theoretical framework and technical systems to support it. The construction of theoretical frameworks and model innovations based on multi-disciplinary and multi-domain land use simulation and prediction has become a trend. The theoretical framework mainly integrates theories and methods of different disciplines, such as geography, ecology, management, environmental science, and sociology. For example, Liu et al. constructed a coupled framework based on genetic algorithms and game theory to coordinate the spatial competition among different land use types [82]. Nie et al. constructed a system framework for tradeoffs in spatial patterns of land use based on the food–energy–water nexus network, revealing the interdependencies and potentially competing interests among the FEW elements in the system, as well as policy, sustainability, and feedback from various stakeholders [83].

In model construction, an important goal is to achieve the sustainability of land use in spatial allocation. For example, Garcían et al. integrated the concept of sustainability in spatial planning to construct a Mauss model, which uses three objective functions, the sub-regional scale (maximizing income, minimizing negative pressure on the environment, and minimizing food deficit) [84]. The model uses the ability of multi-objective evolutionary algorithms. Multiple objective functions are utilized to perform a detailed evaluation of the characteristics of the relevant regions, rather than oversimplifying the spatial analysis to a single capability or fitness function. In addition, learning from research paradigms in diverse fields to improve the operating mechanism of models is often an important way to model innovation [85]. To meet future development needs, it is necessary to improve the accuracy of supply and demand forecasts, the matching of time and space distribution, the convenience of management and operation, and the sustainability of long-term development. These will be an important improvement direction for the theoretical framework and model construction of spatial pattern of land use simulation and prediction.

#### 3.5.4. Adaptive Management of Sustainable Land Use under Multiple Pressures

Global climate change and changes in spatial pattern of land use caused by human activities are the two dominant factors affecting the mechanisms of yield and confluence [86]. The impact of land use changes on climate change and ecosystems and their mutual feedback mechanism is important to global climate change research. To identify the mechanisms and effects of large-scale changes in spatial pattern of land use on the subsurface, affecting the water and heat distribution patterns and energy balance of the land surface, and thus affecting the ecosystem and climate change, a quantitative parametric analysis of the impact of changes in the spatial pattern of land use on climate and ecosystems needs to be performed, which is one of the core scientific problems that must be solved to scientifically understand the impact of spatial land use pattern changes on global climate change [87].

Land use change is not only the cause and consequence of global environmental change but also a necessary means of mitigating and adapting to global environmental change if viewed from another perspective [54]. In particular, sustainable land use management will help reduce the negative impact of different pressures on ecosystems and the economy and will also promote climate change mitigation and adaptation. The way of mitigating CO_2_ emissions through land system management has become an important issue against the background of global warming. Empirical studies have shown that in areas with high-intensity human activities, the optimization of land management measures can increase vegetation carbon sequestration and carbon sinks. This is a more promising way to increase carbon sequestration in terrestrial ecosystems in the future and has more room for replication [22]. Some scholars are concerned about the impact of large-scale new energy development on spatial land use. Coruhlu et al. used a combination of GIS and hierarchical analysis to reveal the solar energy potential of Turkey, providing options for tradeoffs in environmental planning for land use decisions [88]. Cobuloglu et al. studied the competition and tradeoffs between willow branches and corn for biofuel and food production. They obtained the best decisions on land allocation, sowing time, harvest time, quantity, and farm operating budget allocation for food and energy crops to maximize the overall economic and environmental benefits [89]. From the perspective of adaptive management, more attention is paid to rural land use, comprehensive assessment of risks, and land policy transmission mechanisms. Research in this area often involves micro-entities, such as farmers, thus incorporating the enhancement of farmers’ well-being into optimizing the spatial pattern of land use. For example, Cotter et al. (2014) argue that uncertainties and risks in agricultural land use allocation can affect the sustainability of agriculture, and establishing a multi-risk assessment framework for agricultural land use allocation is beneficial for effective assessment of risks under climate change [90]. In the process of transmission mechanism, scholars focus on the land use decision making of farmers against the background of climate change and simulate how farmers’ views on random events change the land use strategy and its financial and ecological results and feedback [91]. For example, Jin et al. conducted a study on the transmission mechanism of land policy simulation based on a land use optimization policy simulation framework model and a system dynamics model to optimize land use management by comparing the applicability of policy simulation [5]. In adaptive management research, the main focus is the high-risk exposure areas of climate change, such as ecologically fragile areas, coastal zones, and urbanized areas of developing countries [92]. At the same time, the way to predict and evaluate future land use changes and their impact on the climate is also a frontier topic in this field.

## 4. Discussion and Perspectives

**Figure 10:**
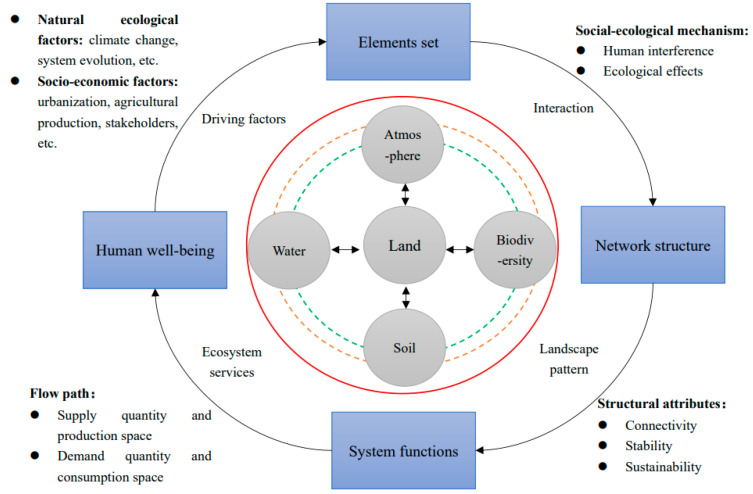
Optimization conceptual model of spatial pattern of land use based on ENSH.

From the above analysis, we find a clear logic of “element sets–network structure–system functions–human welfare” in land use from the perspective of human–land coupling. Therefore, in the context of ecological restoration, we propose an “element sets–network structure–system functions–human well-being” (ENSH) conceptual model for the optimization of spatial pattern of land use based on land system science (Figure 10). That is, the optimization of spatial pattern of land use is taken as an important tool for sustainable socio-ecological system development, and its goal is to achieve the restoration and improvement of the self-organization capacity of the land-centered ecosystem network in order to achieve sustainable land–ecological system function and enhanced human well-being. In this process, it is essential to clarify the socio-ecological mechanisms of spatial land use change, identify and quantify the primary sources of disturbance from human activities and the response thresholds of land ecosystems, and pay attention to the synergistic response mechanisms of socio-ecological landscape networks. We also advocate the inclusion of ecosystem service flows because it is generally believed that not all ecosystem services can be consumed in situ, and spatial displacement from supply to benefit areas must occur; the inclusion of ecosystem service flows can fully understand the trajectories of material and energy change in land ecosystems [93]. It should be noted that the goal of spatial optimization of land use is not simply the pursuit of growth of the potential supply of ecosystem services but also the improvement of human ecological well-being. The ultimate goal of spatial optimization of land use is to realize the improvement and sustainability of transforming ecological elements into ecological well-being in the social–ecological system game process [94]. Finally, the term “safe workspace” is used to connote that the problem of managing the earth’s ecosystem is framed within an acceptable level or “boundary” [95]. Land ecosystem security status should be within the soft boundary, as depicted by the green circle. Instead, it should not exceed the hard boundary depicted by the red circle. Furthermore, the early warning signal should be obtained within the orange boundary.

In order to achieve the goals of the above framework, enhanced research is needed in the following areas.

### 4.1. Concept and Theoretical Framework Innovation

Some scholars believe that land use dimension can be summarized in three levels: space, time, and function, while others believe that land is abstracted from the three significant resources of soil, water, and atmosphere, of which soil is the most basic and objective carrier. It involves ecosystem, landscape, and additional fields [96,97]. At the same time, the spatial optimization of land use is related to the spatial allocation requirements of sustainable land goals, such as ecological mechanism, spatial justice, gender equality, and property rights protection [98]. This requires an appropriate expansion and clear definition of the connotation and optimization of land use, as well as a deeper systematic understanding of the spatial characteristics of land and the composition of its elements from the perspective of human–land coupling. To optimize the spatial pattern of land use under the multi-dimensional integration of social–ecological, multi-level transmission and multi-objective tradeoff, the biggest challenges mainly include supporting the basic theoretical system and constructing a universal methodical system for optimizing the spatial pattern of land use. These are required to encompass not only the reality of land as a form of biophysical change but also the requirements of its multiple sources of value and well-being [99]. Attaching importance to land use spatial pattern optimization and the innovation of the theoretical framework need to change the guidance of simple application of land use spatial pattern optimization research results in practice. The advantages of empirical analysis combined with multi-scale models help expand and innovate the space for land system science to serve practice and help provide a solid basis for a multi-disciplinary intersection that serves land management [10]. Concepts, frameworks, or models that have a firm foothold, in theory, can be used for simple extrapolation as well as for prediction when conditions change substantially [100]. In addition, when the data are scarce or completely missing, they can also be used to test the theory and its impact on empirical data to promote the substantial development of the whole land system science.

### 4.2. Choice of Scale and Time Mechanism

Most of the current research on spatial optimization of land use focuses on finding characteristics within a set scale, “scale characteristics,” without considering the period and spatial range of the essential change of land attributes, “feature scale” [101]. Only by fully understanding its characteristic scale can we find the basic characteristics and the law of evolution. The root of land scale research lies in the heterogeneity of geographical phenomena, the hierarchy of the land system, the nonlinearity of research response and feedback, the influence of interference factors, and the limitation of subjective cognition. Therefore, the selected scale boundary should conform to the principle that there is noticeable feedback within the system, but rather the interaction with the outside of the border is relatively weak [102]. At the same time, with the advancement of globalization, land use decisions on a regional scale are increasingly affected by long-distance markets. The production and consumption of ecosystem services are increasingly spatially separated, forming a complex network of goods and services. It may not be possible to fully explain the driving forces of land use change if we only start from a limited area [57,103]. For this reason, some scholars have proposed the remote coupling of virtual land, land use, and its ecological effects [104]. However, the paths and methods used to effectively integrate the correlation, feedback, and transmission mechanisms between different scales are yet to be improved. The advantages of including ecosystem service flows in the framework include, on the one hand, the identification of key land ecological source sites. On the other hand, it provides the possibility to measure the spillover effects of local land spaces and their cascading effects with landscape patterns.

The integration of the time dimension and the establishment of spatio-temporal explicit optimization models are crucial for the long-term planning practice of sustainable land use [105]. In actual situations, the properties of the plots and the interactions between different land uses are different in space and time, but the current research lacks an in-depth discussion on this [106]. On the one hand, it is necessary to improve the assessment of the time lag inherent in developing land system policies, management changes, and feedback dynamics [107]. On the other hand, the temporal variability and accumulation of land-related parameters need to be fully understood and quantified, and a spatial configuration model over time should be constructed [108].

### 4.3. Revealing the Mechanism of Causal Emergence Theory by Integrating Micro and Macro Scales

Systematically linking socio-economic and biophysical drivers and trajectories is necessary for assessing land use sustainability [109]. On the one hand, there is a need to address the challenge that some social impact factors are difficult to quantify. There is a lack of adequate and systematic ways to bring the policy system, the cognition of different stakeholders, and socio-economic needs into optimizing spatial patterns of land use. For example, Sahraoui constructed the “Actors, Resources, Dynamics and Interactions” ecological network model. Role playing and simulation of diverse stakeholders in various stages of participation in the land use planning program were conducted [110]. Unfortunately, there is a lack of stakeholders and participants to match the demands of urban development based on existing ecological knowledge, and the actual need to improve the overall function of the land ecosystem has not been realized. Qualitative information on the values or preferences of land managers is essential to understand the linkages between drivers, decision-making processes, and the dynamics of land change. Particular attention should be paid to the specific spatial and temporal interdependencies between decision-making processes at different scales, institutional structures, socio-economic contexts, and land system models, which are the deep-seated causes of spatial land use change [10]. It is necessary to deepen the cohesive integration of the impact factors of the natural and socio-economic dimensions to achieve the integration of driving factors of different dimensions and the quantification of interactive feedback. This is helpful to uncover the interaction mechanism among different driving factors, the offset mechanism, the feedback mechanism, and the correlation mechanism between the different driving factors of land use to avoid feedback distortion of the result. The need to quantify the tele-coupling of drivers is urgent, as traditional studies of the drivers of land use change have largely failed to adequately consider the effects of long-range interactions, which increasingly lack explanatory power for land use change driven by long-range interactions in a globalized world [103]. In the framework we constructed, ecological elements, landscape structure, ecosystem services, and human well-being are integrated, and these research themes have a unified theoretical foundation with emergent pathways that reveal the linkages among land use change phenomena from micro to macro scales. Causal emergence theory argues that macroscopic scales can reduce noise in causality, resulting in stronger causal relationships at the macroscopic scale of the system [111].

### 4.4. Identification of Transformation Thresholds

There is a complicated and non-linear dynamic feedback relationship between the spatial pattern of land use and the change of the ecological environment. It is unclear how the direct and chain effects of land use change simultaneously promote ecological change. Moreover, most existing studies assume that there is a one-way process between land use changes and ecological environmental effects, and there are still some difficulties in integrating natural feedback mechanisms into land use change models quantitatively [78]. First of all, the response and feedback mechanisms of the ecological mechanism involve many ecological elements, and they are much more micro. At the same time, the spatial pattern of land use belongs at the macro level, so it is necessary to establish a theoretical bridge to connect the two. The resilience of ecosystems depends mainly on the critical elements of a particular ecosystem. Optimizing these fundamental elements ensures functional diversity and sustainability of natural cycles and processes [112]. Based on the identification of these key elements, it is also necessary to understand the flow paths of these elements and their response thresholds to spatial changes in land use. The functional degradation trajectory of key subsystems should be accurately identified to determine the critical point at which the spatial state of the land changes from green to orange and red. This will indicate the right “window of opportunity” for when and where to implement ecological restoration measures. Only then can we better weigh the relationship between land use and socio-economic development from the perspective of spatial allocation. Of course, this tradeoff also requires a sufficient understanding of the socio-environmental system’s decision-making process and feedback mechanisms to provide a basis for the optimal policy mix [113].

### 4.5. Improving Uncertainty in Simulation and Prediction

The interaction between global environmental change, human activities, climate warming, and change in spatial patterns of land use, and the uncertainty of the direction, rate, and intensity of land use transfer are increasing [114,115]. These are critical realistic factors that cause uncertainty in the simulation and prediction of land use spatial patterns. In addition, uncertainty also runs through the whole land use spatial change simulation and prediction process. Problems with data quality, scale selection, unit division, and weight assignment can lead to uncertainty in simulation prediction results [116]. To cope with these uncertainties, data matching and path identification between the two subsystems of changes in spatial patterns of land use and human activities must be enhanced. Research data from “multiple” to “systematic” should be promoted with the help of big data, artificial intelligence, and other technological advances to realize the effective connection between empirical data, statistical data, and observation data [117]. In terms of modeling, it is necessary to unify data, mechanisms, and models based on deepening the mechanisms of social and ecological processes of spatial changes in land use and recognizing the complementarity of theory, practice, and data. At the same time, the algorithm solving process should be improved to reduce the problem size and integrate the quantitative and spatial structures for consideration [118]. In addition, the reasonable definition of spatial standards, such as connectivity, compactness, and compatibility, as well as a reasonable assessment of spatial relationships, also need to be paid attention to [105]. The integration of a priori stakeholders needs to be emphasized in the simulation forecasting process to avoid an overly mathematical simulation forecasting paradigm. Scenario setting should be based on the understanding of potential futures of infinity and focus on the set of options that are understandable and easy to manage [119], integrating issues of maximizing human well-being, the stochastic nature of policies and stakeholders, and the divide between optimal solutions and operational reality, linking visions of tradeoffs to numerical models that incorporate uncertainty in future projections.

## 5. Conclusions

Spatial optimization of land use has become a key way to improve the function of land ecosystems and thus promote the sustainable development of coupled human–land systems. Based on the current research progress, this review summarizes the research frontiers in this field and puts forward the research challenges and improvement directions. The main conclusions include:

(1) The description of the spatial pattern of land use has transformed from pattern to function and then to well-being. The driving mechanism shifts to both the “expansion effect” caused by rapid economic growth and the “restraint effect” caused by ecological and environmental constraints. To optimize the spatial pattern of land use under the ecological restoration paradigm, ecological elements, processes, and effects have become the focus of attention.
(2) Revealing the socio-ecological mechanisms of spatial changes in land use and elucidating land system network flow paths and response mechanisms have become a research frontier. The research focus of spatial land use simulation forecasting includes improving the accuracy of supply and demand forecasting, matching spatial and temporal distribution, the convenience of management operation, and the sustainability of adapting to long-term development. Research on the role of different stakeholders, response characteristics and adaptation paths, and policy transmission mechanisms is also being strengthened.
(3) Facing the needs for spatial optimization of land use under multi-dimensional integration of society and ecology, the multi-level transmission of scale, and multi-objective tradeoff, we propose to carry out spatial optimization of land under the framework of “element sets–network structure–system functions–human welfare”. Based on the constructed conceptual model, future research needs to strengthen the conceptual and theoretical innovations underlying the accumulation of empirical studies. Meanwhile, it should also focus on the selection of spatio-temporal mechanisms, the revelation of causal emergence mechanisms, the determination of transformation thresholds, and coping with uncertainties in simulation predictions.

## Figures and Tables

**Table 1:** Simulation and prediction models for spatial pattern of land use research.

Types	Representative Models	Main Advantages	Limitations
Spatial models	CLUE-S model, Markov model	The analysis integrates natural and socio-economic factors, spatial and non-spatial, based on the competing land use relationships.	Lack of quantification of processes and effects; failure to consider the matching of various types of economic and social data with land use space.
Planning models	System dynamics model, the linear programing model, multi-objective programing model	It quantifies the social and economic driving factors and the quantitative relationship of their interactions in the complex land use system and determines the supply and demand of regional land use.	Insufficient consideration of the natural properties of land and spatial representation of the results; assumption of a definite causal relationship between land use and drivers.
Simulation and prediction models	Cellular automaton model, FLUS model, genetic algorithm, ant colony algorithm, and particle swarm optimization and simulated annealing algorithm	It can well combine the remote-sensing image data to carry out the spatial description of the micro-mechanism; better definition of the conversion rules of land use space.	Sensitive to the input data; the use of artificial rules instead of human decision making is likely to cause a significant difference between the regularity of the spatial organization structure of the simulation system and the reality, and there is a risk of overfitting.
Intelligent models	Agent-based model, multi-agent models	The decisions and interactions of micro-individuals with dynamism and adaptability are considered in the simulation prediction process.	Too much emphasis on the field of sociology, insufficient attention to the complexity of human society, and easy to ignore the natural-society comprehensive adaptability problem in the process of land change.
Mixed/coupling models	CA–Markov model, the logistic–CA coupling of Markov model	Can give full play to the advantages of each model.	Verifying the accuracy of the results is more complex.

## Data Availability

Not applicable.
